# Rejuvenation of three germ layers tissues by exchanging old blood plasma with saline-albumin

**DOI:** 10.18632/aging.103418

**Published:** 2020-05-30

**Authors:** Melod Mehdipour, Colin Skinner, Nathan Wong, Michael Lieb, Chao Liu, Jessy Etienne, Cameron Kato, Dobri Kiprov, Michael J. Conboy, Irina M. Conboy

**Affiliations:** 1 Department of Bioengineering and QB3, UC Berkeley, Berkeley, CA 94720, USA; 2 California Pacific Medical Center, Apheresis Care Group, San-Francisco, CA 94115, USA

**Keywords:** blood exchange, therapeutic plasma exchange, multi-tissue rejuvenation, rejuvenation by dilution

## Abstract

Heterochronic blood sharing rejuvenates old tissues, and most of the studies on how this works focus on young plasma, its fractions, and a few youthful systemic candidates. However, it was not formally established that young blood is necessary for this multi-tissue rejuvenation. Here, using our recently developed small animal blood exchange process, we replaced half of the plasma in mice with saline containing 5% albumin (terming it a “neutral” age blood exchange, NBE) thus diluting the plasma factors and replenishing the albumin that would be diminished if only saline was used. Our data demonstrate that a single NBE suffices to meet or exceed the rejuvenative effects of enhancing muscle repair, reducing liver adiposity and fibrosis, and increasing hippocampal neurogenesis in old mice, all the key outcomes seen after blood heterochronicity. Comparative proteomic analysis on serum from NBE, and from a similar human clinical procedure of therapeutic plasma exchange (TPE), revealed a molecular re-setting of the systemic signaling milieu, interestingly, elevating the levels of some proteins, which broadly coordinate tissue maintenance and repair and promote immune responses. Moreover, a single TPE yielded functional blood rejuvenation, abrogating the typical old serum inhibition of progenitor cell proliferation. Ectopically added albumin does not seem to be the sole determinant of such rejuvenation, and levels of albumin do not decrease with age nor are increased by NBE/TPE. A model of action (supported by a large body of published data) is that significant dilution of autoregulatory proteins that crosstalk to multiple signaling pathways (with their own feedback loops) would, through changes in gene expression, have long-lasting molecular and functional effects that are consistent with our observations. This work improves our understanding of the systemic paradigms of multi-tissue rejuvenation and suggest a novel and immediate use of the FDA approved TPE for improving the health and resilience of older people.

## INTRODUCTION

Heterochronic parabiosis has been used for decades in laboratory animals to investigate the effects of shared blood, organs and environmental enrichment, on the surgically connected partners [1, 2, 3–5]. The general conclusion of these studies was that the old partners had better health and/or repair of cartilage, muscle, liver, brain, spinal cord, kidneys, bone, skin, etc., and often the young animals experienced premature aging of their respective tissues [1, 3, 4, 6–8]. More recently, blood exchange studies demonstrated that blood heterochronicity alone, without the parabiotic sharing of organs or environment, quickly rejuvenates muscle and liver, but interestingly, not hippocampal neurogenesis or cognitive performance of old mice, and that young mice exhibit rapid and significant decline in hippocampal neurogenesis, agility, learning and liver regeneration after a single exchange with old blood [9].

Historically, the phenomena of heterochronic parabiosis and blood exchange remained unconfirmed with respect to the key assumption as to whether the addition of young factors is needed for rejuvenation, and if premature aging of young mice stemmed from the introduction of old blood factors or a simple dilution of young factors. To answer these questions in a well-controlled experimental set-up, we took advantage of our recently developed small animal blood exchange model [9].

Specifically, we performed a “neutral” blood exchange (NBE) by replacing the platelet-rich-plasma (PRP), fraction with physiological saline, supplemented with 5% purified commercial (fraction V) albumin (e.g. replenishing the depletion of blood albumin). Through a half-hour long series of small volume exchanges, 50% of the PRP of old and young mice was replaced with saline plus 5% mouse albumin while the circulating red and white blood cells were returned isochronically to the animal.

Young (2-4 months) and old (22-24 months) male C57/B6 mice underwent a single NBE; and, as a control for the procedure, we performed isochronic blood exchanges: young – young (YY) and old – old (OO) [9]. Subsequent analyses of muscle repair, liver adiposity and fibrosis, hippocampal neurogenesis and serum proteomics were all done at 6 days after a single NBE.

The procedure of small animal blood exchange is time consuming and labor intensive: blood vessels of mice are cannulated and checked for patency, the next day animals are checked for catheter patency again, and then undergo the exchange under general anesthesia. Of note, two mice are used for each NBE where one mouse provides freshly derived synchronic syngeneic blood cells, which are resuspended in saline + albumin, for the second animal that undergoes the NBE. We thus calculate the minimal number of experimental animals that is needed for statistically significant conclusions and allow for feasible experimental work.

A common error is to base the statistical significance of an observation on whether the sample size is large. The statistical significance for independence between samples or a measurement of statistical power (Power) is not solely dependent on the number of samples (N) but is measured through the effect size (ES) and Variance.

The metric used to determine the amount of statistical power in normal distributions is defined as:

$$\begin{array}{c} X\sim N(0,1):\mu =0,\sigma^{2}=1\;(\text{normal,}\;\text{i}\text{.i}\text{.d}\text{.}\;\text{assumption})\\ n=\text{sample}\;\text{size},\;\hat{\sigma }=\text{sample}\;\text{variance},\;\alpha =0.05\\ S_{n}^{\left(1\right)}=\left\{s_{1},s_{2},\ldots ,s_{n}\right\},\;S_{n}^{\left(2\right)}=\left\{s_{1},s_{2},\ldots ,s_{n}\right\}\;\\ \text{ES}\;(\text{effect}\;\text{size})=\frac{1}{n}\left(\sum_{i=1}^{n}S_{n}^{\left(1\right)}-\sum_{i=1}^{n}S_{n}^{\left(2\right)}\right)\\ \text{critical}\;\text{valus}=\left|\text{X}\text{.ppf}(\frac{\alpha }{2})\right|=\left|\Phi^{-1}(\frac{\alpha }{2})\right|\\ \text{Power}=1-\text{X}\text{.cdf}\left(\text{critical}\;\text{value}-\frac{\text{ES}}{\hat{\sigma }/\sqrt{n}}\right) \end{array}$$

**Figure 1:**
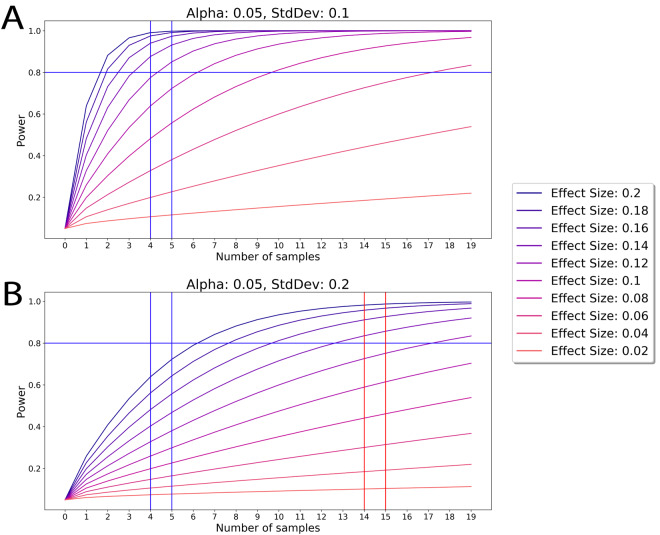
**The Power of an experiment is determined by the Effect Size.** (**Top**) According to the ES and Variance in comparing young, old and old rejuvenated cohorts in our 2003-2020 studies we show that with these parameters N=4 justifies independence in our samples and larger N does not significantly improve on this justification. (**Bottom**) If hypothetical samples have high variance (and thus normalized effect size is reduced), then more samples (higher N) are needed to procure similar power in an experiment. Generally, an increase of sample size is needed for increasing the Power to discern less-tangible phenomena that are not statistically detectable without such N increase.

In Figure 1 below, we simulate power by modulating effect size and plotting these against the number of samples. A power of 0.8 is a high threshold standard of robust studies; lesser power is often used in published work and more than 0.8 power comes at a disproportionate cost of resources and time (diminishing returns of logistic function) [10, 11]. As shown in Figure 1, when the Variance of independent experiments is low and the difference in ES between the cohorts is high, e.g. as in our 2003-2020, including published and current studies on young, old and rejuvenated old cohorts [1–3, 4, 9] there is no difference in Power between N=4, 8, 19, or infinity. In contrast, in the absence of robust phenotypes, if two phenomena are detected as the same or have very modest effect, with high enough N the statistical power will strictly increase. Of note, this approach applies only to theoretical data obtained with no loss in precision, no detraction from statistical power and no preference. In the real-world this can permit not just detection of weak phenomena, but also overfitting and accumulation of artifacts that are misinterpreted as biological phenomena at some higher values of N.

Based on the above analysis and preliminary YY, OO, YNBE and ONBE work with N=3 for each cohort, a Power of 0.8 and higher and robust significance of our conclusions was obtainable with N=4. We set up 4 of each isochronic YY and OO exchanges, which is a control repeat of the same studies (exchange process and analyses of muscle, liver, and hippocampal neurogenesis) that we previously published with 8 of YY and OO mice [9]. These were done in parallel with the new NBE studies. In work on repair of injured muscle, we performed four independent characterizations of adult myogenesis: regenerative index, fibrosis, minimum Feret diameter and myogenic proliferation with data agreement between all and between the mouse and human studies. In liver studies, we performed two characterizations of liver health: adiposity and fibrosis, latter was assayed by two independent methods; the data agreement was seen in 8 independent start-to-finish experiments with each cohort. In the studies of hippocampal neurogenesis, we performed 6-7 independent start-to-finish experiments with each cohort and these are in agreement with our in vitro studies with neural precursor cells. Each assay on each sample and tissue was done in multiple replicates. Additional tissues’ samples were used for work on other ideas and hypothesis (in progress). Of note, recent studies, comparable in scope and methods, published in high impact journals, have N =3-4 with even when the ES is lower, and Variance is larger than that of current work [12].

**Figure 2:**
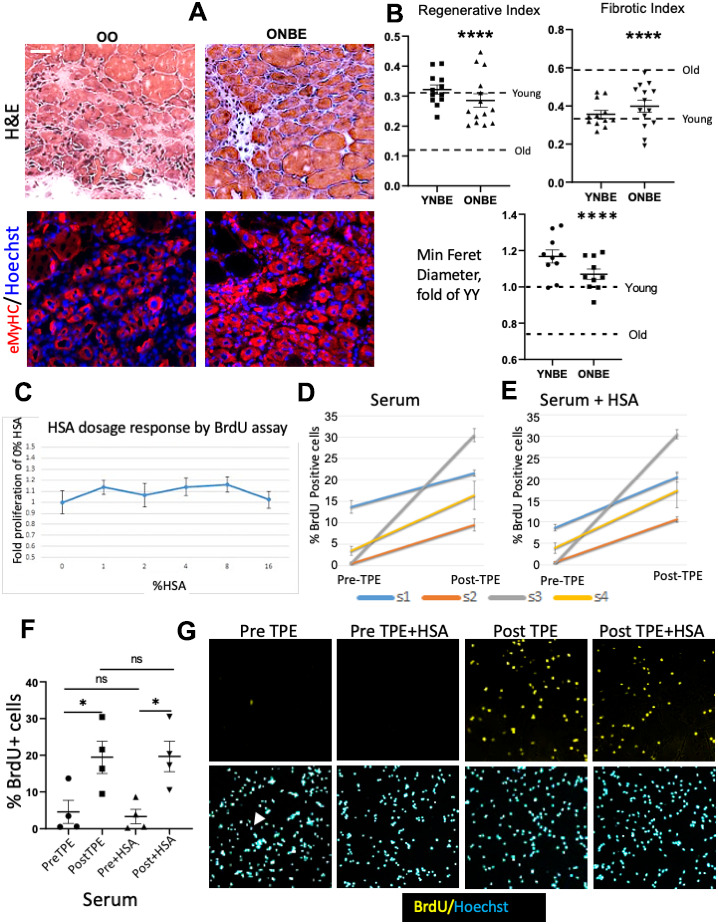
**Rejuvenation of adult myogenesis, and albumin-independent effects of TPE.** One day after the NBE, muscle was injured at two sites per TA by cardiotoxin; 5 days later muscle was isolated and cryosectioned at 10 μm. (**A**) Representative H&E and eMyHC IF images of the injury site. Scale bar = 50 μm. (**B**) Regenerative index: the number of centrally nucleated myofibers per total nuclei. OO vs. ONBE p = 0.000001, YY vs ONBE non-significant p = 0.4014; Fibrotic index: white devoid of myofibers areas. OO vs ONBE p = 0.000048, YY vs YNBE non-significant p = 0.1712. Minimal Feret diameter of eMyHC+ myofibers is normalized to the mean of YY [9]. OO vs. ONBE p= 3.04346E-05, YY vs. YNBE p=0.009. Data-points are TA injury sites of 4-5 YNBE and 5 ONBE animals. Young and Old levels (detailed in Supplementary Figure 1) are dashed lines. Representative images for YY versus YNBE cohorts are shown in Supplementary Figure 6. **(**C**) Automated microscopy quantification of HSA dose response, as fold difference in BrdU+ cells from OPTI-MEM alone (0 HSA). There was no enhancement of myogenic proliferation at 1-16% HSA. N=6. (**D**) Meta-Express quantification of BrdU+ cells by automated high throughput microscopy for myoblasts cultured with 4% PreTPE versus PostTPE serum and (**E**) for these cells cultured with 4% of each: PreTPE serum + HSA or PostTPE serum + HSA. Significant increase in BrdU positive cells is detected in every subject 1, 2, 3, and 4 for TPE-treated serum (p=0.011, <0.0001, <0.0001, 0.0039, respectively), as well as for TPE-treated serum when 4%HSA is present (p<0.0001, <0.0001, <0.0001, =0.009 respectively). N=6. (**F**) Scatter plot with Means and SEM of all Pre-TPE, Post-TPE, +/- HSA cohorts shows significant improvement in proliferation in Pre TPE as compared to and Post TPE cohorts (p*=0.033), as well as Pre+HSA and Post+HSA cohorts (p*=0.0116). In contrast, no significant change was observed when comparing Pre with Pre+HSA (p=0.744) or Post with Post+HSA (p=0.9733). N=4 subjects X 6 independent assays for each, at each condition. (**G**) Representative BrdU IF and Hoechst staining in sub-regions of one of the 9 sites that were captured by the automated microscopy. Blood serum from old individuals diminished myogenic cell proliferation with very few BrdU+ cells being visible (illustrated by one positive cell in Pre-TPE and arrowhead pointing to the corresponding nucleus); TPE abrogated this inhibition but HSA did not have a discernable effect.

Muscle repair was assayed on the success in formation of new myofibers that replaced injury sites and the degree of fibrosis, both quantitatively assayed by hematoxylin and eosin (H&E) staining, and minimum Feret diameter of newly-formed eMyHC+ muscle fibers was quantified, as we previously published [1, 6, 7, 9, 13, 14]. YY muscles regenerated much better than OO, as expected [1, 9, 14, 15]. Interestingly, a single NBE improved regeneration, reduced fibrosis and increased minimum Feret diameter of de-novo myofibers in old mice to the point of no significant difference with the young, and NBE did not worsen these attributes of young muscle repair, Figure 2A, 2B. Muscle fibrosis was not different between YY and young control mice that did not experience blood exchange or OO and old control mice; regeneration was slightly better in YY than Y and in OO than O cohorts; and YY/Young cohorts had higher regeneration and lower fibrosis than OO/Old cohorts (Supplementary Figure 1).

To confirm these findings and to explore their evolutionary conservation, we took advantage of the fact that there is a procedure for human patients analogous to NBE, where most of the plasma is replaced by physiologic solution supplemented with commercial human albumin, called Therapeutic Plasma Exchange, TPE, which is FDA approved and routinely used in the clinic [16–18]. And so, we studied human blood samples, which were collected Pre and Post TPE, from four old individuals (65-70 years of age). We previously established that mouse primary myoblasts can be used for determining the effects of the proteins that are secreted by human cells on myogenic cell proliferation and differentiation [15, 19, 20]. Here we performed BrdU-based cell proliferation assay in C57.B6 primary myoblasts, which are over 95% pure as per Pax7/Myf5/MyoD markers ([13] and Supplementary Figure 1). These muscle progenitor cells were cultured in OPTI-MEM with 4% PreTPE versus PostTPE human serum for 20 hours in triplicate wells of two replicate 96 well plates (6 replicates total for each cohort, 15,000 cells per well). BrdU was added for the last 6 hours of culture, after which cells were fixed, immuno-detection of BrdU was performed, and high throughput automated microscopy was used for imaging 9 sites of each well and for subsequent data quantification.

As demonstrated in Figure 2, old human serum strongly reduced myogenic cell proliferation (which is consistent with the effects of the old mouse serum [1, 2, 6, 7, 13]). Interestingly, single TPE procedure removed this inhibition and allowed for robust proliferation of myogenic progenitors that were cultured with the PostTPE old serum, e.g. from the same individual(s) whose PreTPE serum was strongly inhibitory.

The positive control for myoblast proliferation (BrdU assay on cells in Growth medium, GM), no-serum control (OPTI-MEM) and the negative control for non-specific fluorescence (BrdU assay on myoblasts in GM that were not treated with BrdU) are shown in Supplementary Figure 1 that demonstrates robust myoblast proliferation in GM, good proliferation in OPTI-MEM and confirms that BrdU immunofluorescence was highly specific.

To start dissecting the possible mechanisms by which exchange of old mammals with saline plus albumin exerts these rejuvenative effects, we examined whether ectopic albumin (human serum albumin, HSA) might be a determinant. First, we performed HSA dose curve, which demonstrated that ectopic albumin does not promote myoblast proliferation (Figure 2F). Based on this dose-curve, we added 4% HSA to our myoblast proliferation assay (where cells were cultured with 4% Pre versus Post TPE human serum). Interestingly, while there was consistently better myoblast proliferation with PostTPE serum as compared to the PreTPE, HSA neither rescued the proliferation of PreTPE myoblast cultures, nor added to the proliferation of the Post-TPE cultures (Figure 2).

Western Blotting confirmed that serum albumin does not decrease with age (Supplementary Figure 2), and there is no net gain of albumin in NBE/TPE, but rather back-supplementation of the procedure depleted serum, albumin. In people, albumin levels correlate with disease, nutrition, and socio-economic status rather than chronological age; and even when health, etc. status are not considered, albumin diminishes only marginally, by 2-4% at 75 years of age from its 26 years of age levels [21–24].

We also looked at the antioxidant properties of PreTPE versus PostTPE serum. Albumin has antioxidant activity [16], and thus we tested if Post TPE serum might promote myogenic cell proliferation simply through improved antioxidant properties. Interestingly, we did not find antioxidative difference between Pre and PostTPE serum (Supplementary Figure 3).

These results establish that NBE and TPE have positive effects on adult myogenesis and that ectopic albumin does not rescue the old serum-imposed inhibition of myogenic proliferation.

In the original work on rejuvenation of old animals and aging of young through heterochronic parabiosis, the effects were shown not only for skeletal muscle, but also for the liver and brain-hippocampal neurogenesis [1, 7]. The hippocampal neurogenesis data was reviewed in Nature in 2004 [7] but it was not published in 2005 [1]; nevertheless, it was repeated and published in Nature in 2011 [25]. Similarly, the effects of heterochronic blood exchange were studied with all these three germ layers derived tissues [9]. Hence, we performed the analyses of hippocampal neurogenesis and liver health (degree of adiposity and fibrosis) to examine whether in addition to skeletal muscle, there might be rejuvenative effects in the absence of young blood.

**Figure 3:**
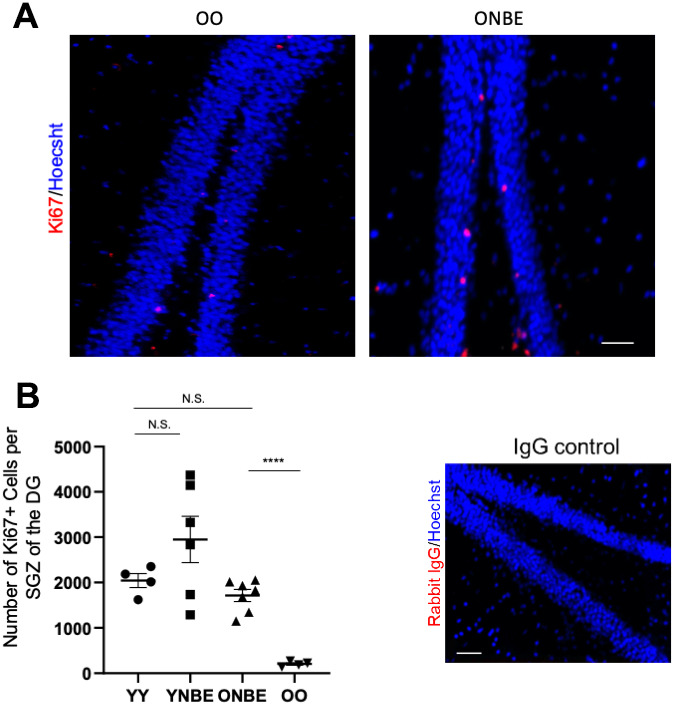
**Neurogenesis of aged mice is enhanced by one procedure of neutral blood exchange.** (**A**) Immunofluorescence was performed to assay for proliferative Ki67-positive cells in the subgranular zone (SGZ) of the dentate gyrus (DG). Representative images of Ki67(red)+/Hoechst (blue)+ cells in the DG are shown for OO and ONBE mice. (**B**) Quantification of the number of Ki67+/Hoechst+ cells per SGZ of the DG (extrapolated from serial sections that span the entire hippocampus). ONBE mice have a ~8-fold increase in the number of these cells when compared to OO (****p-value = 0.0000145). The number of these proliferating neural precursor cells in the SGZ of YY mice is not significantly different from that of ONBE mice (N.S. p-value = 0.15235). A trend for ~44% increase in YNBE mice as compared to the YY mice, is not statistically significant (N.S. = 0.20123). Isotype-matched IgG negative control confirms low non-specific fluorescence. N=4 for YY and OO, N=6 for YNBE and N=7 for ONBE. Scale bar is 50-micron. Representative images for YY versus YNBE cohorts are shown in Supplementary Figure 6. These data demonstrate that hippocampal neurogenesis improves in old mice after just one NBE, e.g. without young blood or its fractions, and that young mice do not decline in this parameter when their blood plasma is diluted through the NBE.

Neurogenesis in the subgranular zone (SGZ) of the hippocampus continues throughout adult life, dramatically declines with advanced age and is influenced by blood heterochronicity [7, 9, 14, 26]. To assay the effects of NBE on SGZ neurogenesis, proliferation of the neural precursor cells in the SGZ of Dentate Gyrus was quantified throughout the thickness of hippocampi, using immuno-detection of the proliferation marker KI67 in serial brain cryosections (Figure 3A, 3B). YY mice had ~10-fold higher numbers of Ki67+ SGZ cells than old, as expected [9, 15, 26] and notably, there was a significant and large ~8-fold increase in hippocampal neurogenesis in old mice after a single NBE, with no statistical reduction of hippocampal neurogenesis in young mice (Figure 3A, 3B).

This increase was larger than what was observed after heterochronic parabiosis or through defined pharmacology of modulating the TGF-beta and oxytocin pathways [15]. SGZ localization of proliferating cells was used, as typically done, for determination of their neural precursor cell identity [27]; Ki67 was nuclear and non-specific immunofluorescence of isotype-matched IgG controls was negligible (Figure 3 and Supplementary Figure 6.

To continue the study with ectopic albumin, we performed BrdU proliferation assay with neural precursor cells, NPC, which provide a good in vitro correlation to the efficiency of hippocampal neurogenesis [15, 28]. Interestingly, in contrast to the lack of effects on myogenic cells, ectopic albumin enhanced NPC proliferation by itself – in the absence of serum and improved NPC proliferation when old serum was present in the cultures (Supplementary Figure 3B). These results agree with previously published enhancement of proliferation of retinal precursor cells by albumin [29] and with efficient proliferation of human iPSC-derived NPCs on electrospun serum albumin fibrous scaffolds [30].

However, the body of published work consistently demonstrates that albumin is a negative factor for brain health. With respect to reaching neural cells in vivo, Blood Brain Barrier becomes leaky with age [31, 32] and serum albumin crosses it and is found in cerebro-spinal-fluid of older individuals in a positive correlation with age and with certain types of dementias [33]. Moreover, in direct test, infusions of albumin into the brain were deleterious: causing neuro-inflammation, excessive TGF-beta1 and neuronal dysfunctions [34].

In our work the total levels of albumin were not altered, but rather replenished from those diminished by NBE/TPE, thus negative effects on the brain are not anticipated.

To expand our data in muscle (mesoderm) and brain (ectoderm) to an endodermal tissue lineage, and to compare all with the previous experiments on heterochronic parabiosis [1, 7] and heterochronic blood exchange [9], we studied liver adiposity and fibrosis in NBE vs. isochronically blood exchanged mice. Liver adiposity and fibrosis are known to increase with age and to be influenced by blood heterochronicity [1, 9, 14, 35–38].

**Figure 4:**
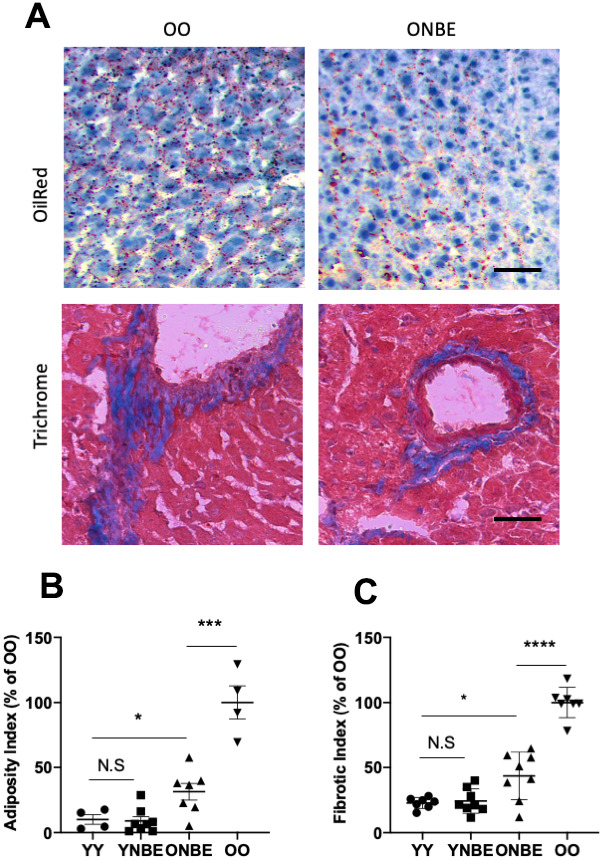
**Liver adiposity and fibrosis are reduced in old mice after a single procedure of neutral blood exchange.** Histological analysis (Oil Red-O and Masson’s trichome staining) of 10 μm liver sections from uninjured mice collected at 6 days after NBE. (**A**) Representative images of lipid droplets (fat) stained with Oil Red-O and Collagen (fibrosis) stained blue with Masson’s trichome show that NBE visibly reduced fat and fibrosis of old livers. (**B**) Adiposity Index (red pixels per section) and (**C**). Fibrotic index (numbers of fibrotic clusters per section) were determined as in [9] and by shown here trichrome. Adiposity: YY-YNBE NS p= 0.8, YY-ONBE *p= 0.04, OO-ONBE ***p= 0.0004. N=4 YY and OO, N=8 YNBE and ONBE. Fibrosis: YY-YNBE NS p= 0.7, YY-ONBE *p= 0.012, OO-ONBE ****p= 0.00001. N=8. All quants are represented as % of OO control. Scale bar=50 μm. Representative images for YY versus YNBE cohorts and of albumin/Hoechst are shown in Supplementary Figure 6.

Liver adiposity, or fat in liver, was assayed by conventionally used Oil Red staining Figure 4, [9, 14, 28, 39] and liver fibrosis was assayed by Albumin/Hoechst immunofluorescence, as published [2, 14] and shown in Supplementary Figure 6, and additionally, by Masson’s trichome (Figure 4). Adiposity and fibrosis were higher in the OO livers as compared to YY, as published [1, 9, 14]. Interestingly, after the NBE these parameters of liver health became much improved in the old mice, and did not worsen in the young mice (Figure 4).

These results show that NBE can supplant for the positive effects of young blood on old liver health, and that a dilution of young blood does not diminish the studied liver health attributes in young mice.

Summarily, these results establish broad tissues rejuvenation by a single replacement of old blood plasma with physiologic fluid: muscle repair was improved, fibrosis was attenuated, and inhibition of myogenic proliferation was switched to enhancement; liver adiposity and fibrosis were reduced; and hippocampal neurogenesis was increased. This rejuvenation is similar to (liver) or is stronger than (muscle and brain) that seen after heterochronic parabiosis or blood exchange.

These findings are most consistent with the conclusion that the age-altered systemic milieu inhibits the health and repair of multiple tissues in the old mice, and also exerts a dominant progeric effect on the young partners in parabiosis or blood exchange.

**Figure 5:**
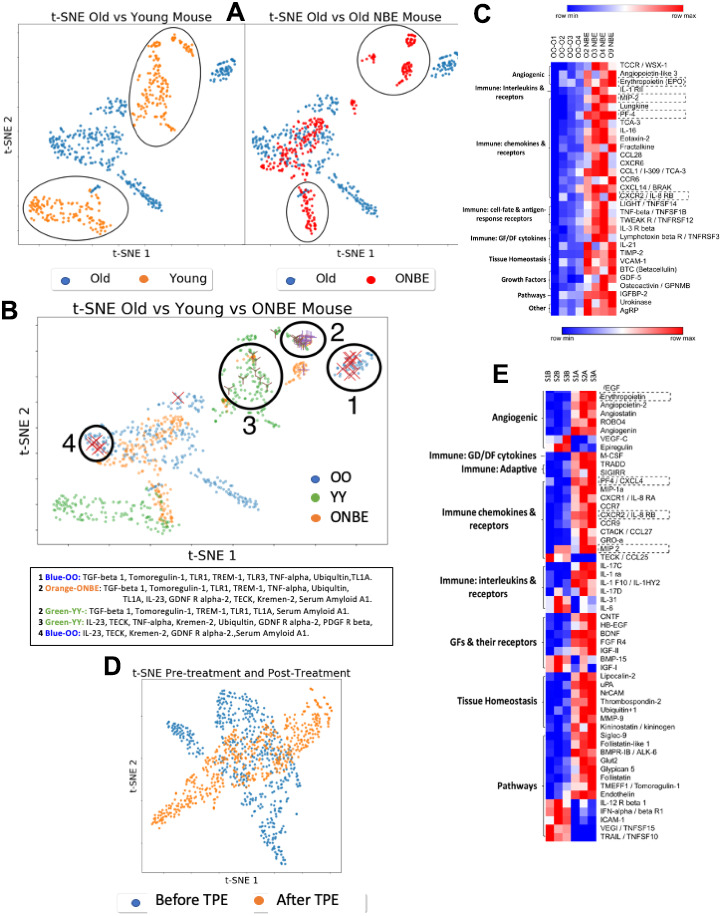
**Comparative NBE/TPE proteomics.** Serum levels of 308 mouse proteins (Raybiotech #L-308) and 507 human proteins (Raybiotech #L-507) were assayed. After background subtraction, total intensities for each protein (assayed in duplicates for each sample) were normalized to the internal array background control as fold-increments, with a sensitivity cut-off of 2-fold; these normalized intensities were expressed, as a fraction of the internal array positive control. (**A**) t-SNE clustering of mouse proteins grouped by class of treatment: OO and YY isochronic controls were compared to each other (left) and OO was compared to ONBE (right). Differences between YY vs. OO and OO vs. ONBE proteomes are outlined. (**B**) Distinct grouping of OO, YY and ONBE proteomes is shown in the t-SNE plot with identities of proteins in clusters 1-4 specified below. Power analysis for independence of X, X and Y marked proteins from these clusters, is shown in Supplementary Figure 4. (**C**) Heatmap on mouse proteins illustrates significant differences between OO and ONBE cohorts (proteins are grouped on their main function, as indicated). (**D**) Old human serum proteome before TPE (Before-B) and 1 month after a single procedure of TPE (After-A): t-SNE clustering of human proteins grouped by class of treatment. TPE resulted in a clear and robust change in the molecular composition of the systemic milieu as compared to the Before-TPE. (**E**) Heat map on human proteins illustrates significant differences between S1,2,3 B and S1, 2, 3 A (before versus after TPE) cohorts (proteins are grouped on their main function, as indicated). Proteins in dashed boxes are the same between mouse and human in Heatmaps. A general elevation (not decrease) of most systemic proteins at 6 days after the NBE and at 1 month after the TPE was observed.

To address the molecular mechanism of this rapid rejuvenation of all three germ-layers derived tissues, we performed comparative antibody array proteomics on the mouse and human serum that were derived before versus after the NBE/TPE, as well as YY and OO mouse serum (Figure 5). Proteins of low, medium and high levels are detected with high sensitivity (high dynamic range) in this set-up, as demonstrated by Figure 5, Supplementary Figure 4, and Supplementary Table 1.

Multi-dimensional t-SNE analyses and Heatmapping of these data revealed that the ONBE proteome became significantly different from OO and regained some similarities to the YY proteome (Figure 5A–5C). Supplementary Figure 4 confirms the statistical significance of this comparative proteomics through Power Analysis, and shows the YY vs. OO Heatmap, where the age-specific differences are less pronounced than those between OO vs. ONBE, again emphasizing the robust effect of NBE on the molecular composition of the systemic milieu.

As true for the mouse data, human O Before TPE was also robustly different from O After TPE, establishing a significant change in the composition of human circulatory proteins one month after a single procedure (Figure 5D, 5E; and Supplementary Table 1). In an evolutionary-conserved fashion in both mice and humans, angiogenic regulators, immune regulators, and growth factors (including neurogenic ones), were changed in their systemic levels. A few NBE/TPE changed proteins were the same in both species. Very interestingly, even though we diluted blood serum, many of the NBE/TPE modulated proteins were up-regulated (Figure 5, Supplementary Figure 4 and Supplementary Table 1), suggesting that their production or secretion was inhibited by the age-elevated systemic factors in both mice and in people.

An age-associated increase in VCAM-1 was recently suggested to account for the negative circulatory effects on the brain [40]. We observed similar levels of VCAM-1 (p=0.953) between young and old serum which was confirmed by Western Blotting (Supplementary Figure 5), and an elevation (not decrease) in VCAM-1 after the NBE, e.g. when old tissues in mice were rejuvenated (Figure 5, Figures 2–4). Considering that there is little change in VCAM-1 in blood with age, it is unlikely that NBE acts through youthful normalization of this cell-surface protein, which might be shed into the circulation. TPE there was no significant change in VCAM-1 versus before the procedure (p= 0.072675), and similarly, we did not see a difference in CCL11 [25]. Of note, another cell-surface protein was implicated in brain aging, B2M [41], the invariant chain of the MHC class I molecule, which is indicative of broadly diminished inflammation [9, 42]. We have shown that systemic levels of B2M do not increase in the circulation of old mice as compared to young; and an exchange of old mice with young blood diminishes the tissue levels of B2M [9].

This work shifts the paradigm of blood heterochronicity away from dominance of young blood factors and establishes that replacement of a large volume of old blood with a neutral age physiological fluid (saline supplemented with 5% purified albumin), is sufficient for most if not all observed positive effects on muscle, brain and liver. Importantly, it shows that a currently approved FDA procedure promotes molecular and functional rejuvenation of the blood in older people, with improved proteomic profile and support for myogenic responses.

Promising work in progress follows an interesting observation that TPE anecdotally reduced incidents of viral diseases to zero in patients over the course of a year when for just flu-related hospitalizations, ~60% were from the same age group (https://www.cdc.gov/flu/about/season/flu-season-2017-2018.htm). TPE has the potential to improve recovery from viral illnesses and diseases, particularly for older people, through a number of ways, including changes in cytokine, interleukin, growth factor profiles and enhanced immunity, and restoration of virus-diminished oxytocin receptor [43] via productive attenuation of TGF-beta pathway [14]. Better myogenesis, angiogenesis and tissue vascularization in the old are also suggested by our data, hence better overall organ health and repair, better success of vaccination is predicted.

Our pilot longitudinal study with samples from several old individuals highlights the most robust effects of TPE that are observed even with this small sample size, providing insights for future work, in the classic tradition of case and pilot studies. Phase 2B and Phase 3 clinical trials are being actively developed; these will expand our findings and extend these to vaccination outcomes and improved recovery from viral illnesses.

This study does not exclude the possibility that factors that are present in young blood, as well as sharing of young organs and environmental enrichment, might also contribute to the rejuvenative effects of heterochronic parabiosis. It is yet to be determined whether and to what degree NBE/TPE also improve neuronal differentiation, neuroplasticity and cognition, metabolic health more generally than liver adiposity and fibrosis, as well as the health and maintenance of other tissues that have been reported rejuvenated in heterochronic parabioses.

Interestingly, a single NBE does not significantly worsen the examined parameters in young mice; albeit, a higher variation between YNBE mice is detected for all the studied tissues, as compared to the YY cohort. Thus, young animals might be able to offset, at variable individual degrees, one dilution of their platelet-rich-plasma (possibly, upregulating gene expression for a compensatory elevation of protein production and/or secretion). A higher myofiber diameter was detected in YNBE as compared to YY, which might reflect differences in myogenic proliferation and/or differentiation.

**Figure 6:**
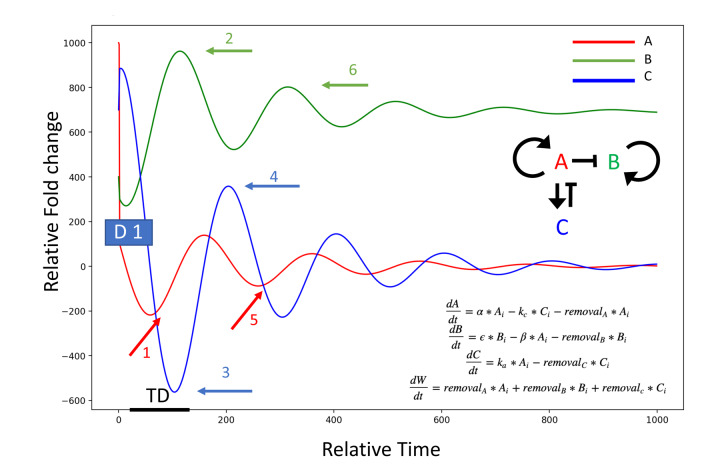
**Model of the dilution effect in resetting of circulatory proteome.** System: A induces itself (A, red), and C (blue); A represses B (green), C represses A. A dilution of an age-elevated protein (A, at D1: initial dilution event), breaks the autoinduction and diminishes the levels of A (event 1, red arrow); the secondary target of A (B, at event 2 green arrow), then becomes de-repressed and elevated (B induces B is postulated); the attenuator of A (C, at event 3 blue arrow), has a time-delay (TD) of being diminished, as it is intracellular and was not immediately diluted, and some protein levels persist even after the lower induction of C by A. C decreases (no longer induced by A), and a re-boot of A results in the re-induction of C by A (event 4 blue arrow) leading to the secondary decrease of A signaling intensity/autoinduction, and a secondary upward wave of B (events 5 red arrow and 6 green arrow, respectively). *alpha* = 0.01, k_c_ = 0.01, *beta* = 0.05, *epsilon* = 0.1, *k_a_* = 0.1. Protein removal rates from system: *removal_A_* = 0.01, *removal_B_* = 0.1, *removal_C_* = 0.01, Initial values: *initial_A_* = 1000, *initial_B_* = 400, *initial_C_* = 700.

The functional phenotypes and comparative systemic proteomics suggest some evolutionary conservation and help to explain the rapid and profound resetting of the systemic milieu accompanied by tissue rejuvenation through exchange of old blood with saline-albumin. We simulate this in Figure 6, where many systemic age-elevated proteins, such as the TGF-beta family [7, 14, 15 44–47], are known autoinducers, and where dilution will diminish their expression and feedback will decrease the levels for a prolonged time [48–56]. Consequentially, their secondary targets – other proteins whose expression was inhibited in the old (such as “young” factors needed for tissue maintenance and repair) become derepressed and increase (as we indeed observe for numerous proteins); and entire pathways (MAPK, for example) are no longer hijacked for non-canonical induction of TGF-beta, Smad1, and inflammation [57, 58], but instead engage in productive signaling by their cognate ligands, promoting tissue health, regeneration and homeostatic production of cytokines and growth factors [59–61] (with their own positive feed-backs), as we observe. When the levels of ligand(s) diminish, attenuators also diminish, and receptor(s) increase [62, 63]; signaling re-boot induces negative regulators, as we observed for follistatin and follistatin-like, secondarily diminishing the intensity of age-elevated pathways [64–66]. With variable feedbacks and protein half-lives, changes in every autoinductive protein, secondary target, receptor, etc. are not expected to be simultaneously detected at any given time point, but many are indeed observed at 6 days post NBE and 1 month after TPE.

The above concept fits well with the age-imposed increase in systemic TGF-beta family ligands (GDF11 and TGF-beta 1, for example), which contributes to pro-geronic phenotypes [7, 14, 15 44–47] and the fact that attenuation of TGF-beta signaling in old animals has effects that are similar to those of NBE [7, 14, 15, 28, 44]. NBE is also predicted to promote stronger rejuvenation than an Alk5 inhibitor, as that attenuates just one branch of one pathway, and because proteins other than the TGF-beta family that are elevated with age will be re-set to their younger levels of gene expression and/or signaling intensities by NBE/TPE (to be profiled in the future). Fitting the model that is shown in Figure 6 with experimental data on multiple time points after NBE/TPE, for multiple proteins and multiple levels of regulation (mRNA, protein, signaling intensities), is a focus of our long-term work.

It is also quite possible that multiple mechanisms contribute to the rejuvenation of the three germ layer tissues by NBE, with the above described model being just one. For example, while we did not see an effect in myogenesis, ectopic albumin might promote enhanced immunity in NBE/TPE, particularly after multiple in vivo procedures. There was a positive effect of albumin on NPC proliferation, which agrees with published results; and yet delivery of ectopic albumin worsened brain health, and CSF albumin is a marker of brain aging and disease. Overall, it does not seem that albumin is the only determinant of NBE/TPE, but it might have a contribution particularly when the age-elevated factors become diluted.

Of note, we grouped the proteins loosely, as many of them are pleiotropic and play a role in more than one functional group. For instance, lipocalins are homeostatic transporters of lipophilic molecules but also play a role in productive innate immunity [67], and IL-8 receptor beta, also known as CXCR2, plays a role in immune responses, angiogenesis and has a cross-talk with PI3K, p38/ERK, and JAK/Stat signaling pathways [68].

With respect to the proteins that are the same between mice and people, and were modulated in the same direction by the NBE and TPE, an increase in erythropoietin is likely to improve the numbers and health of erythrocytes, attenuating age-imposed anemia [69]. MIP2 controls migration of neutrophils to sites of inflammation, and an increase could help to resolve inflammaging [70]. PF4 promotes platelet aggregation, it is broadly chemotactic, plays a role in wound repair and has anti-microbial activity [71]. IL-8 receptor signaling promotes cell survival, migration, chemotaxis, angiogenesis, oligodendrocyte positioning, and might attenuate neuropathy [72].

Looking at the general protein categories, an increase of angiogenic regulators is expected to broadly contribute to improved vascularization [73, 74] and through this increased perfusion of tissues, to tissue repair. Immune regulators promote better surveillance and wound clearance in multiple tissues [75, 76]. Increases in neurotrophic growth factors BDNF and CNTF, and of GDF5 which broadly and positively regulates formation of cartilage, brown fat, and neuronal axons and dendrites, have implications for better maintenance and repair of damage in the CNS and periphery.

Modulation of multiple key cell-fate regulatory pathways, including attenuation of pSmad2,3 signaling by follistatin, is important for productive cell proliferation and regeneration in skeletal muscle, heart and generally, throughout the body [77–79]. And, follistatin-like has broad anti-inflammatory properties [80], which are also expected to promote better health broadly in a mammal. Similarly, a general positive effects are expected from the elevation by TPE of Tomoreguilin-1, which antagonizes Nodal and Junk signaling, was shown to combat cardiac hypertrophy [81], to be needed for CNS formation and maintenance [82], and to be neuroprotective, by binding toxic Alzheimer’s Disease amyloid beta [83].

Finally, improved tissue maintenance is typically accompanied by a healthy remodeling [77, 84, 85], which makes it of significance that MMP9, VCAM-1, TIMP2, and thrombospondin are being modulated by the NBE and/or TPE. Of note, while we observed an increase of TIPM2 after an NBE (Figure 4), e.g., in agreement with more active tissue remodeling, at the baseline there was no age-specific change in TIMP-2 (p=0.217 YY vs OO).

Summarily, the focus on the *before* versus *after* systemic proteomics defined the key changes that correlate and logically fit with the rapid and robust rejuvenation of multiple tissues. It is possible that many of the observed molecular systemic changes are simultaneously required for the enhancement of maintenance and health of old tissues, or alternatively, smaller groups of these proteins might suffice for multi-tissue or tissue-specific rejuvenation.

The theoretical significance of this study is in a better understanding of how blood heterochronicity acts to quickly and profoundly rejuvenate old mammals, and the clinical significance of this work is in developing TPE as a new modality to broadly improve organ health and repair in older individuals preventing illnesses that develop or become more severe in later decades of life.

## MATERIALS AND METHODS

### Animals

All *in vivo* experiments and procedures were performed in accordance with the policies set by the Office of Laboratory Animal Care and under the approved protocol. Young male C57BL/6 mice (2 months old) were purchased from Jackson Laboratory while aged mice (18 months old) were purchased from the National Institute of Aging.

Blood exchanges were performed as in [9]. Briefly, mice were pre-anesthetized by buprenorphine and anesthetized with isoflurane in oxygen to full relaxation. Ophthalmic ointment was applied to each eye to prevent drying. A 1Fr to 3 Fr heparinized catheter (Instech Labs, C10PU-MJV1403) was inserted into the jugular vein and the caudal ligature became tightened to hold the catheter in place. Once patency has been confirmed, an additional cranial ligature is made to latch the catheter in place. Antibiotic ointment with lidocaine was applied to the site. The mice were taken off anesthesia and dosed with subcutaneous Meloxicam (5 mg/kg s.q.) for 7 days post-procedure. Blood from young or aged donor mice was obtained by terminal cardiac puncture and anticoagulated with 3 units of heparin. Blood samples were centrifuged spun at 500g for 5 minutes. The platelet rich plasma fractions were carefully removed, blood cell pellets were resuspended in normal saline and then spun down once more at 500 g for an additional 5 minutes. The saline layer was removed, and blood cell pellets were then resuspended in an equal volume of 5% MSA in normal saline, 0.9% sodium chloride. The replacement albumin is >95% pure by manufacturer’s analysis (https://mol-innov.com/products/albumin-mouse-plasma/). These blood mixtures were passed through a 50um FACS mesh cap tube in order to de-clump cells and filter out any clots. Extracorporeal blood exchanges were performed between pairs of young mice, pairs of old mice, and young mice or old mice and a tube containing synchronic blood cells in 5% MSA several hours following the surgeries. These exchange procedures were performed in a similar fashion as previously published [9].

### Experimental muscle injury

Tibialis anterior (TA) muscles of mice were injured by intramuscular injections of cardiotoxin (CTX; Sigma Aldrich). 10 μL of CTX were injected per TA at 0.1 μL/mL. TA’s were isolated five days post injury.

### BrdU assay was performed as in [15, 20]

On shelf samples of human blood (collected through the FDA approved and routinely used in the clinic procedure of Therapeutic Plasma Exchange [16–18]) were provided to us by Dr. Dobri Kiprov (IRB Approved by Sutter health, 648823) without personal identifiers and were not linked to or traced back to any personal information in our study. Human blood work was done under the approved biological use authorization protocol

Muscle satellite cell-derived primary C57.B6 myoblasts, were plated in OPTI-MEM with 4% human serum on Matrigel-coated Greiner clear-plastic 96-well plates at 15,000 cells/well for 20 hours. Human serum Albumin was added to some wells at 4%. BrdU was added for the last 6 hours of culture. Cells were fixed with 4% PFA for 5 minutes and 70% EtOH for 15 minutes. 3M HCl was used for 30 minutes. Blocking was done using 1% BGS in PBS (staining buffer) for 1hr. Cells were incubated with Rat anti-BrdU primary antibodies at 0.5ug/ml in PBS,1%BGS overnight at 4C; and then, after 3x washes, with secondary antibodies (goat anti-rat) at 0.5ug/ml and Hoechst at in PBS, 1%BGS for 1.5hrs at room temperature. Cells were washed 3x with PBS it each buffer change step. Cells were imaged with Image Xpress Micro (IXM) such that images were acquired at 9 sites per a well of 96 well plates. Quantification was done using MetaXpress for percent BrdU positive cells (TRIT-C) out of all cells (Hoechst). One outlier with very low cell numbers, e.g. one well (out of 6) for one sample was removed from the data analysis. ImageJ was used for equal adjustments of brightness and contrast of representative images.

Rat neural precursor cells were cultured on chamber slides in basal medium with the indicated percent of old mouse serum and/or purified mouse serum albumin (5% stock solution in saline, e.g. “4% albumin” is 4% of a 5% solution). Cultures were acclimated during the day and BrdU added overnight, then cultures were fixed in 4% paraformaldehyde followed by 70% ethanol. Samples were immunostained for BrdU using acid antigen retrieval and % BrdU+ve cells were counted and estimated by two blinded researchers, with the results averaged.

### Tissue isolation

Mice were sacrificed per the guidelines of UC Berkeley’s OLAC administration. Blood was collected by terminal cardiac puncture and was allowed to clot at room temperature over 30 minutes. Blood serum for proteomics analysis was obtained by centrifuging clotted blood samples at a speed of 5,000 g for 5 minutes. Postmortem isolation of muscle, liver, and brain was performed. Tissues were embedded in tissue-tek optimal cutting temperature (OCT, Sakura Finetek, The Netherlands) and snap frozen in isopentane cooled to -70°C with dry ice.

### Tissue sectioning

OCT-embedded tissues were sectioned with a cryostat. Muscle and liver were sectioned to 10 μm thickness while coronal sections of brain were obtained at 25 μm. Tissues sections were attached to gold-supplemented positively charged glass coverslip slides in preparation for immunofluorescence or histological analysis.

### Hematoxylin and eosin staining

H&E staining was performed as previously published [9, 14]. Slides with mounted muscle tissue sections were dehydrated in 70% ethanol for 3 min and then 95% ethanol for 30 seconds. Tissues were re-hydrated in deionized water for 1 min, and then placed in hematoxylin for 5 min and 1X Scott’s water for 1 min. Slides were rinsed in water for 1 min, treated with eosin for 4 min, and rinsed in water again for 30 s. A final dehydration series of 70%, 95% twice, 99 and 100% ethanol, for 1 min each was performed. Sections were washed with xylenes twice, 1 min each. 2 drops of 50% resin/50% xylenes mounting medium were added to each slide and glass cover slips were placed. Injury sites were imaged accordingly.

### Antibodies and labeling reagents

The following antibodies were used at 0.5 – 1 μg/mL:

- Albumin: R&D Systems, Mouse, MAB1455, 1:1000 for immunofluorescence, 1:2000 for Western blotting
- αRat-BrdU (Abcam ab6326)
- Embryonic Myosin Heavy Chain: F1.652 clone, Developmental Studies Hybridoma Bank, University of Iowa, deposited by Blau, HM) 1:100
- Ki67: Abcam, Rabbit, ab16667, 1:200
- Sox2: R&D Systems, Mouse, MAB2018, 1:50
- VCAM1: Abcam, Rabbit, ab134047, 1:2000
- Isotype-matched IgG’s: Sigma Aldrich, Mouse and Rabbit, 1:500 and 1:1000 respectively
- Donkey anti-mouse Alexa 488: Life Technologies, Invitrogen, Eugene, Oregon, A21202, lot #1975519, 1:^20^00
- Goat anti Mouse IgG (H+L) Secondary Antibody, HRP, Invitrogen #62-6520, 1:2000
- Goat anti-Rabbit IgG (H+L) Secondary Antibody, HRP, Invitrogen #65-6120, 1:2000
- Donkey anti-rabbit Alexa 546: Life Technologies, Invitrogen, Eugene, Oregon, A10040, lot #1946340, 1:2000
- Goat anti-Rat 546, Abcam ab150165.

Hoechst dye was used to stain DNA: Hoechst 33342, Sigma Aldrich (B2261), 1:1000

### Tissue section immunofluorescence

All tissue sections for immunofluorescence studies were mounted on positively charged Gold super frost slides.

### Muscle

10 μm-thick muscle sections were blocked in 1% staining buffer (1% calf serum in 1X PBS) for 45 minutes at room temperature without fixation or permeabilization. Samples were coated in primary antibodies and incubated at 4°C overnight. The slides were rinsed in staining buffer and then coated in secondaries for 2 hours at room temperature the following day. Note: muscle sections stained for embryonic myosin heavy chain (eMyHC) were not fixed nor permeabilized; they were blocked as specified and directly treated with anti-eMyHC antibodies.

### Liver

10 μm-thick liver sections were fixed in 70% ethanol overnight and washed with 1X PBS the next day. Liver sections were permeabilized with 0.1 Triton X-100 for 5 minutes on ice, blocked with staining buffer for 45 minutes at room temperature, then incubated with primary antibodies overnight at 4°C. Samples were rinsed with staining buffer and coated in secondaries as noted.

### Brain

25 μm-thick brain sections were fixed with 4% paraformaldehyde (PFA) for 4 minutes in the dark at room temperature. The sections were washed in copious amounts of 1X phosphate buffered saline (PBS) and then permeabilized with 0.1% Triton X-100 for an additional 5 minutes over ice. Samples were washed with thoroughly with 1X PBS and blocked in 1% staining buffer for 45 minutes at room temperature. The brain sections were incubated with primary antibodies overnight at 4°C. The following day, sections were washed three times with staining buffer and coated with secondary antibodies as described.

### All samples

2 droplets of Fluoromount (Sigma F4680) mounting media were added and coverslips were placed on each slide after a final staining buffer wash proceeding secondary antibody and Hoechst incubation. A Zeiss Axioscope fluorescent microscope was used for imaging.

### Oil Red-O staining

Oil Red-O staining was performed as previously described [9, 14]. 10 μm liver sections were rehydrated in 1X PBS for 5 minutes. These sections were washed in 60% isopropanol for 15 minutes, placed in an Oil Red-O staining solution that is isopropanol-based for 15 minutes, and finally rinsed once more in 60% isopropanol for 5 minutes. A one-minute wash in hematoxylin stained the nuclei in these liver sections. A final deionized water wash was performed. 2-3 drops of fluoromount mounting medium were added to the slides, cover slips were placed, and samples were imaged.

### Mason Tri-chrome staining

Masson’s Trichome staining was performed accordingly to the manufacturer’s protocol. Briefly, 10 μm liver sections were fixed in Bouin’s solution for 1 hour at 56 C, then stained in Weigert’s iron hematoxylin for 10 min, in Biebrich scarlet-acid fuchsin for 15 min, in a phosphomolybdic-phosphotungstic solution for 15 min, followed by aniline blue solution for 10 minutes, and rinsed in 1% acetic acid solution for 5 minutes. Liver sections were finally dehydrated in 70%, 95%, and 100% ethyl alcohol and cleared in xylene. 2-3 drops of fluoromount mounting medium were added to the slides, coverslips were placed, and samples were imaged.

### Antibody array proteomics

Mouse Serum levels of 308 proteins (L-308 glass array, RayBiotech, Norcross, GA, USA) and human serum levels of 506 proteins (L-506 array) were measured following manufacturer’s instruction. Briefly, equal volumes of respective serum sample were dialyzed, biotinylated and re-dialyzed. The array membranes were treated with blocking buffer for 1h, followed by overnight incubation at 4°C with biotinylated serum sample. After washing, the arrays were incubated with Streptavidin-conjugated Cy3 fluorescence dye for 2h at room temperature. Finally, the dried glass slides were scanned using GenePix 4000B scanner from Molecular Devices to obtain fluorescence intensities and acquire images of respective arrays. Microsoft Excel was used for data analysis. After background subtraction, total intensities for each protein were normalized to negative control as fold-increments with a cut-off of 2 folds as significant increase. Finally, respective intensities were normalized to positive control to compare against different samples in respective groups.

### Western blotting

Mouse serum samples were prepared by diluting the samples 1:10 and 1:100 in 1X Laemmli and heating the samples to 95 °C for ten minutes. 10 μl of each sample was loaded onto precast 4–20% TGX gels (Bio-Rad) and transferred to 0.45 μm polyvinylidene difluoride membranes (Millipore). To block the membrane, it was incubated in PBS with 0.1% Tween-20 and 2% bovine serum albumin for one hour. Primary antibodies (mouse anti-VCAM-1 and rabbit anti-albumin) were diluted 1:2000 in PBS with 0.1% Tween-20 and 2% bovine serum albumin and incubated overnight at 4 °C. Horseradish peroxidase (HRP)-conjugated secondary antibodies (goat anti-rabbit and goat anti-mouse) were diluted 1:2000 in PBS with 0.1% Tween-20 and 2% bovine serum albumin and incubated 1 hour at 25 °C. Blots were developed using Western Lightning Plus-ECL reagent (Perkin Elmer), and analyzed with a Bio-Rad Gel Doc/Chemi Doc Imaging System and Quantity One software.

### For albumin blotting

2 μL of each serum sample was diluted into 18 μL of 1X Laemmli buffer and heated to 95 deg C for 10 minutes. 1 μL from each of the heated samples was dissolved in 9 additional μL of 1X Laemmli buffer. The 10 total μL of the serially diluted samples were loaded per lane to be probed for albumin (1:100 total sample dilution). A Ponceau red stain was performed and the blots were imaged. Membranes were blocked for over 3 hours in 5% non-fat dry milk in 1X PBST at 4 deg C and then probed for mouse anti-albumin (1:2000) in 5% non-fat dry milk overnight in the cold room. After 3 rinses of PBST, 10 minutes per rinse, the membranes were treated with goat anti-mouse HRP secondary antibodies (both 1:1000) in 1% BSA for 2 hours in the cold room. Samples were rinsed with PBST again, treated with ECL reagent, and imaged.

### Antioxidant activity assay

De-identified patient blood was collected to BD serum tubes, noting only the date, subject number, round of TPE and whether it was collected before or after TPE. Blood was allowed to clot at 37C for 1 hour, and RBCs sedimented by centrifugation at 500xg for 20 minutes. Serum was aliquotted and frozen at -70 until use. Assays were performed as described (Koracevic 2001, doi 10.1136/jcp.54.5.356, https://www.ncbi.nlm.nih.gov/pmc/articles/PMC1731414/ Briefly, 10 microliters of serum were assayed in triplicate and compared with a dilution series of uric acid as standard activity, and absorbance of reacted thiobarbituric acid was measured at 532nm.

### Data quantification and statistics

Data was analyzed using Student's *t*-tests (two-tailed) and P values equal or lower than 0.05 were considered statistically significant. Sample sizes of *n* = 4 or greater were determined for each experiment based on Power Analysis, IACUC considerations, and based on Effect Size, Variance and N that yielded Power of 0.8 in previous experimental studies (2005-2020).

### Quantification of immunofluorescent images for BrdU incorporation

Was performed as in [15]; briefly, nine 10X images were taken on the Molecular Devices ImageXpress Micro automated epifluorescence imager for each replicate well, followed by automated cell quantification using the multiwavelength cell scoring module within the MetaXpress analysis software.

### Tissue histology and immunofluorescence

Muscle regeneration indices were calculated by counting the frequency of centrally nucleated de-novo myofibers relative to the total nuclei in 2-4 representative images per 10 μm muscle section for each cohort. Muscle fibrosis was quantified by measuring areas of fibrosis within muscle injury sites. These obtained fibrotic areas were normalized to the total area of injury site. Neurogenesis was quantified by counting the number of Ki67+/H+ cells in 200 μm of the SGZ from each mouse from multiple as previously described [9]. Fibrotic indices in the liver were quantified in 10 μm sections. The total measured areas of albumin-negative fibrotic clusters or Tri-chrome blue clusters were normalized to the total area of liver tissue present in each image per cohort. Oil Red-O quantification was done by measuring the total area of red fatty droplets in liver sections. These red areas were normalized to the total area of liver tissue in each image per cohort as well. All analyses were performed at 20x magnification. Non-paired, two-tailed t-tests were performed in Microsoft Excel for all tissue analysis data.

### Western blotting

Results were quantified using Image J software. Pixel Intensity of the VCAM-1 bands were normalized with the pixel intensity of albumin, and a non-paired, two tailed t-test was used to compare cohorts.

### Serum proteomics t-SNE plots

The analysis of the 308 plasma proteins measured in mice with t-distributed stochastic neighbor embedding (t-SNE) projection, with perplexity of 40. The t-SNE analysis was also performed on 506 human plasma proteins, with perplexity of 40. The manifold learning package of Python-SciPy was used to perform the t-SNE analysis, and Python-Matplotlib was used to visualize the clustering results.

### Serum proteomics: array data analysis and generation of heat maps

Mouse and human antibody array data were analyzed in Excel 2016. Proteins were identified using the two-tailed Student’s t-test (paired for human data, heteroscedastic for mouse) with p < 0.05. Identified proteins were then sorted by mean fold-change from greatest to least. The heat maps were generated using the free online heat map generator MORPHEUS found at https://software.broadinstitute.org/morpheus/

### Data availability

All relevant for this study data is to be provided during submission.

## Supplementary Material

Supplementary Figures

Supplementary Table 1
