# Supplementary material for: Rejuvenation of three germ layers tissues by exchanging old blood plasma with saline-albumin
Source: Aging (Albany NY). 2020 May 30;12(10):8790–819. doi: 10.18632/aging.103418 (PMC7288913; doi:10.18632/aging.103418)
Supplement: Supplementary Figures [file aging-12-103418-s002..pdf]

SUPPLEMENTARY FIGURES

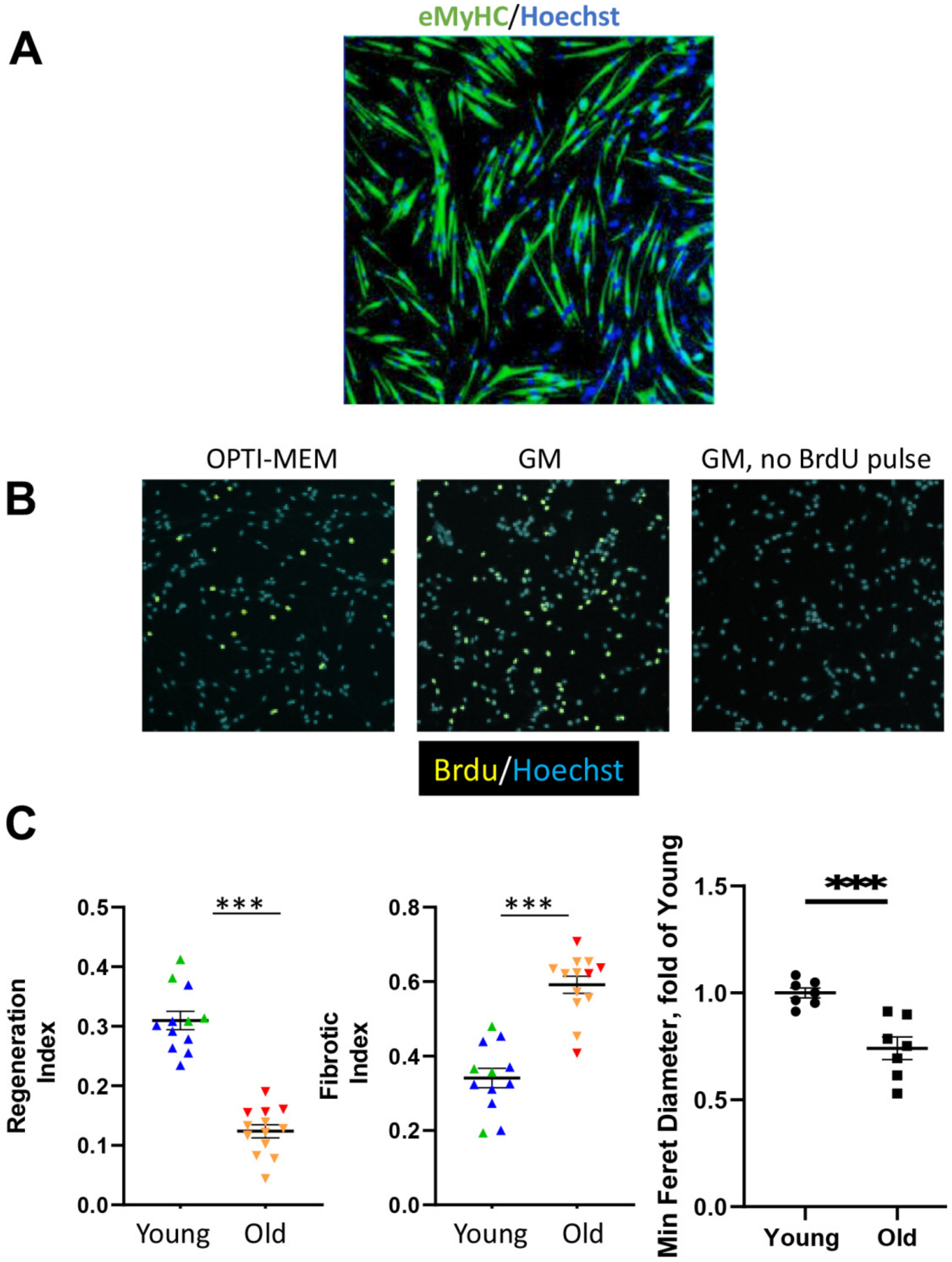

Supplementary Figure 1. Additional controls for BrdU assay and for the assays on Regenerative, Fibrotic indices and Minimal Feret Diameter. (**A**) Primary mouse myoblasts are functionally pure myogenic cells as shown by their robust differentiation into myotubes after 3 days in fusion medium (DMEM, 2% horse serum); cultures were immunostained for eMHC (green) with Hoechst to stain nuclei. (**B**) BrdU/Hoechst overlays of representative automated microscopy generated images of the primary myoblasts in OPTI-MEM (no serum, no HSA), GM (Ham'sF10, 20%FBS, 5ng/ml bFGF) – positive control for efficient myogenic proliferation, and in GM with no BrdU pulse – negative control for immunofluorescence. (**C**) Fibrotic Indices of YY (green) and OO (red) cohorts are not different from those of Young control (blue) and Old control (orange) not-apheresed cohorts, YY vs. Y p=0.8420, OO vs. O p=0.9552; Regenerative Indices are slightly (~10%) higher in YY than Y p=0.03 and in OO than O p=0.004. YY/Y muscle regenerates better than OO/O Reg Index: p=8.26103E-10, Fib Index: p=2.70616E-07; Minimal Feret diameter: YY (Young) vs. OO (Old); p = 0.0007.

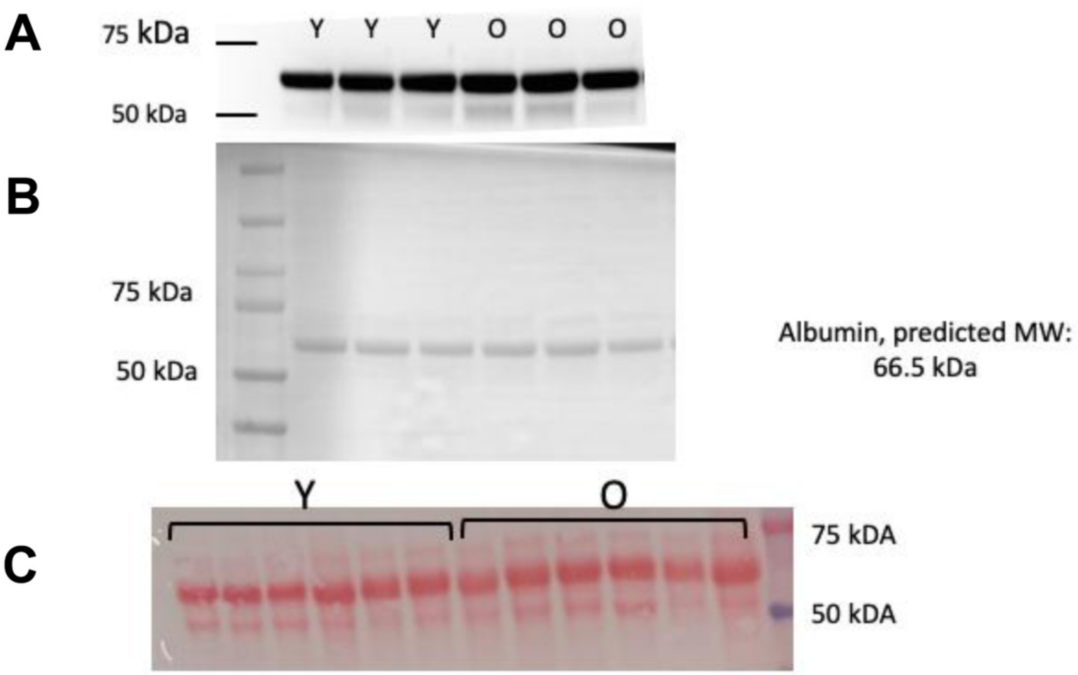

Supplementary Figure 2. Levels of serum albumin do not decline with age. (**A**) Immunoblot for serum albumin, with corresponding Ponceau Red (**B**); and Ponceau Red stain for one more blot (**C**) all demonstrate that there is no age-specific decline in serum albumin. 1 microliter of serum was diluted 1/100 in Laemli Buffer and 10 microliters of that diluted sample was loaded per lane.

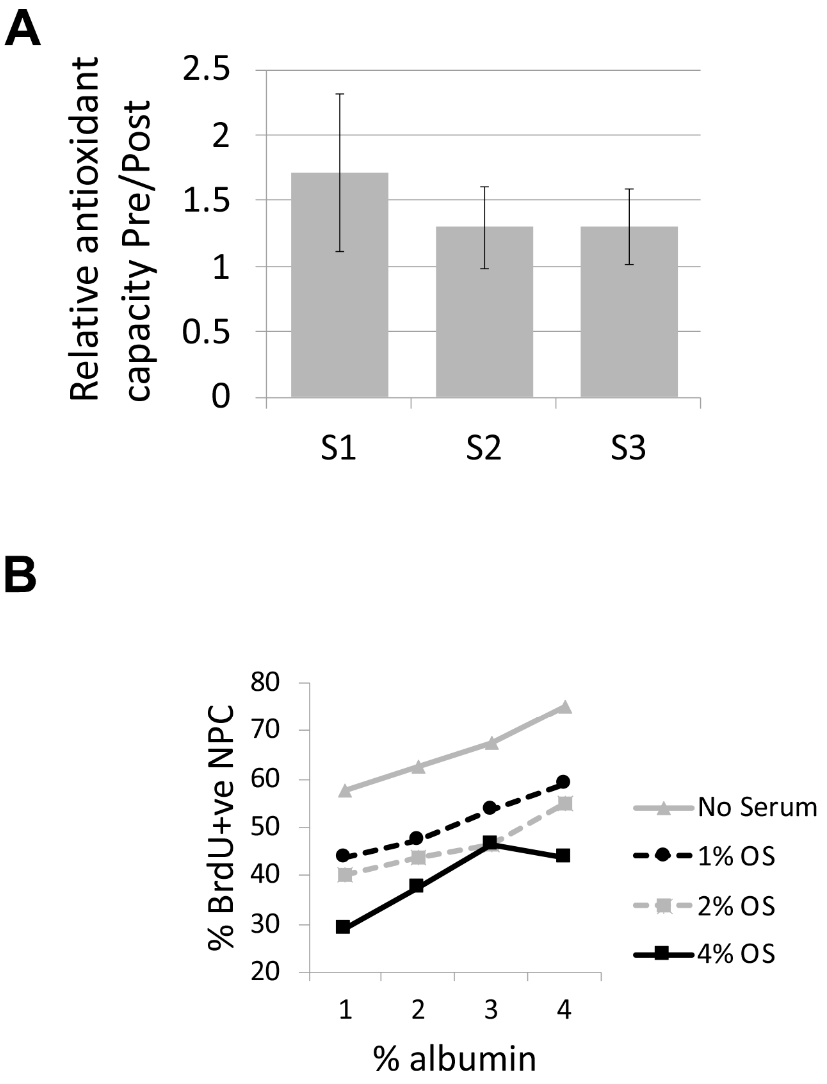

Supplementary Figure 3. Antioxidant activity and effects of albumin on proliferation of NPCs. (**A**) Antioxidant capacity of human blood serum does not appreciably change post TPE. Blood serum samples from three aged healthy volunteers (s1, s2, s3), were collected Pre and Post TPE and analyzed for antioxidant capacity. Plotted are the median values Pre/Post of 5 replicate experiments with standard deviation bars shown. A value of 1 would indicate no change; there is a slight trend of higher activity Pre compared with Post TPE, but the z-test for difference with a value of 1 is 0.03 or less. (**B**) Rat neural precursor cells (Peltier et al., 2010), were cultured on chamber slides in basal medium with the indicated percent of old mouse serum and/or purified mouse serum albumin. BrdU added overnight, then cultures were fixed in 4% paraformaldehyde followed by 70% ethanol and immunostained for BrdU; and % BrdU+ve cells were counted by two blinded researchers, with the results averaged.

A

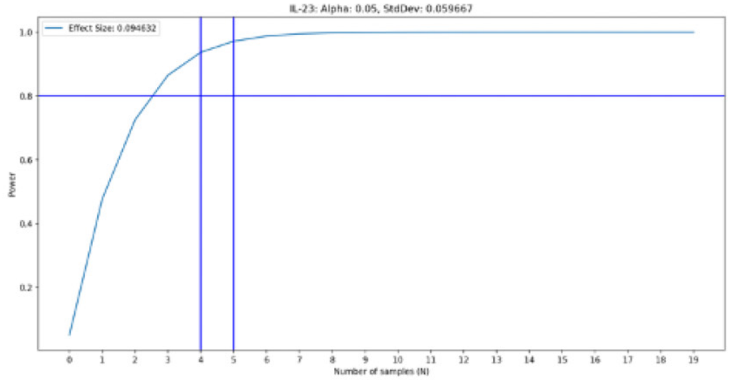

B

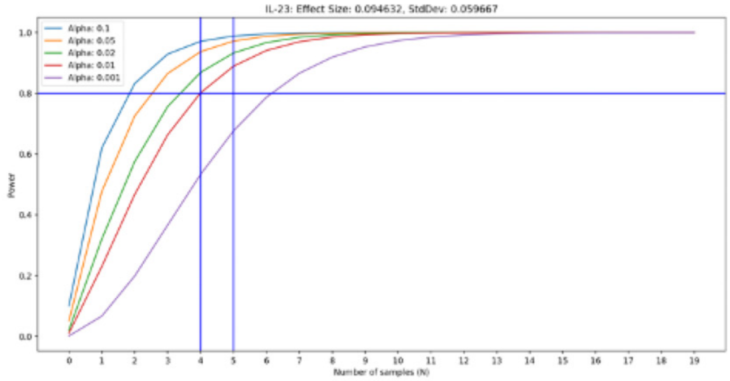

C

	ES	Pooled Stddev
GFR alpha-2 / GDNF R alpha-2	0.087724	0.058523
IL-23	0.094632	0.059667
Kremen-2	0.082423	0.057379
PDGF R beta	0.143614	0.138269
Serum Amyloid A1	0.076952	0.070770
TECK	0.093327	0.052230
TGF-beta 1	0.025581	0.021643
TL1A / TNFSF15	0.084278	0.035148
TLR1	0.034712	0.030832
TLR3	0.046827	0.026903
TMEFF1 / Tomoregulin-1	0.045021	0.029627
TNF-alpha	0.058927	0.050653
TREM-1	0.033235	0.029407
Ubiquitin	0.048449	0.044492

D

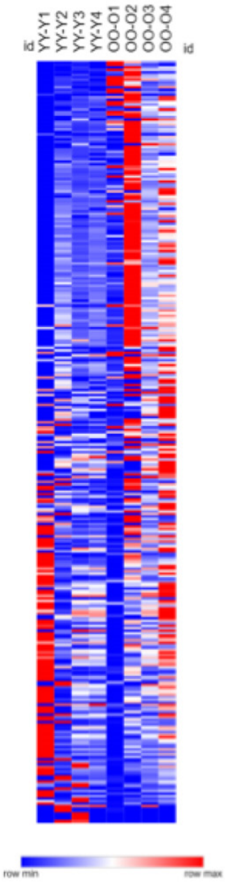

Supplementary Figure 4. Power Analysis and additional information for the comparative proteomics studies. (**A**) As an example, in our proteomics data, IL-23, has a mean effect size of 0.095 and standard deviation of 0.06. With an alpha value of 0.05, we show that 4 samples are sufficient to procure statistical power of over 0.8. (**B**) Even with lower p-values (alpha), we can still see that our effect size for IL-23 is statistically significant. (**C**) Effect Size and Pooled StdDev of the key proteins that shifted from their OO positions to ONBE positions on t-SNE plots. (**D**) All 306 proteins (named and ordered, as in Excel NBE) are shown as a heat map: individual variation between animals appears more visible than an age-specific pattern.

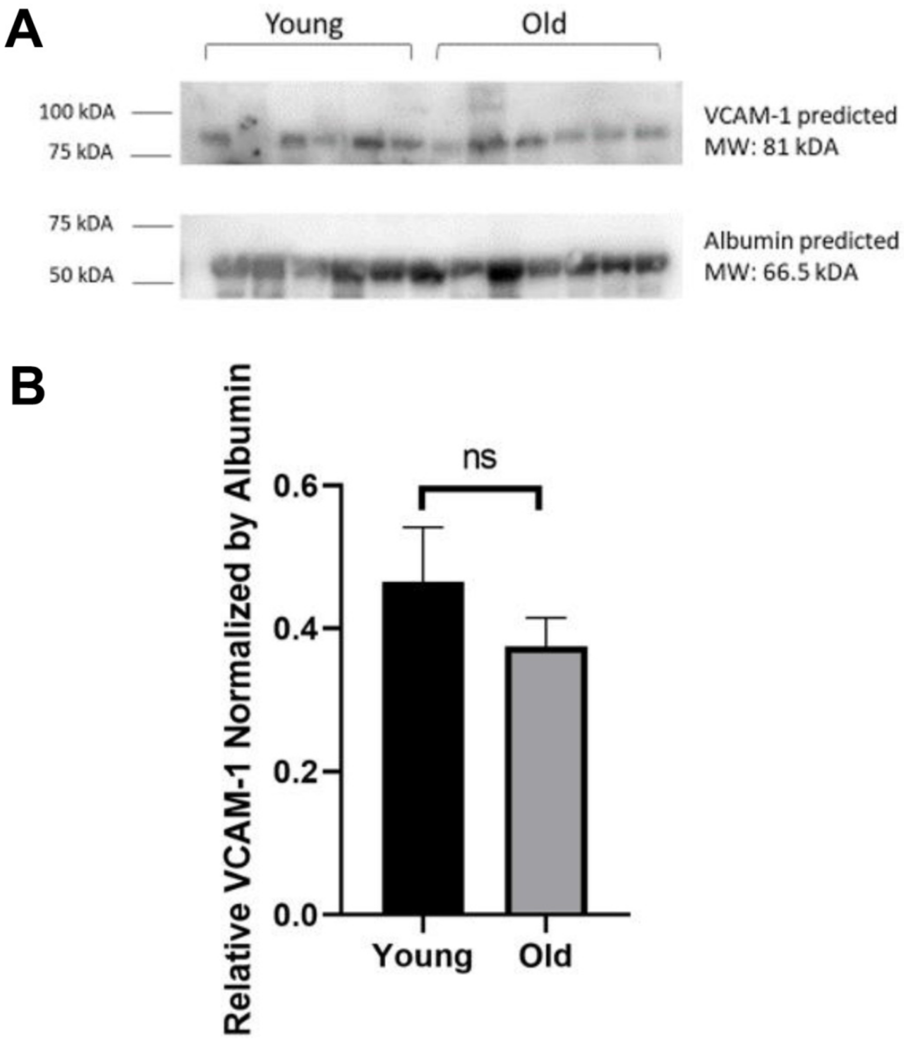

Supplementary Figure 5. VCAM-1 levels in sera do not change between old and young mice. (**A**) Western Blotting with a VCAM-1 specific antibody and albumin as a loading control. Sera was diluted 1:10 in 1X Laemmli for VCAM-1 and 1:100 for albumin. Comparing old and young mice, no discernable difference between the two groups was detected. (**B**) Quantified results: means ± s.e.m. of the pixel intensity of the VCAM-1 bands, normalized with the pixel intensity of albumin. N=6 per group. Two tail student t-test showed no significant differences between the two groups. P=0.315118099.

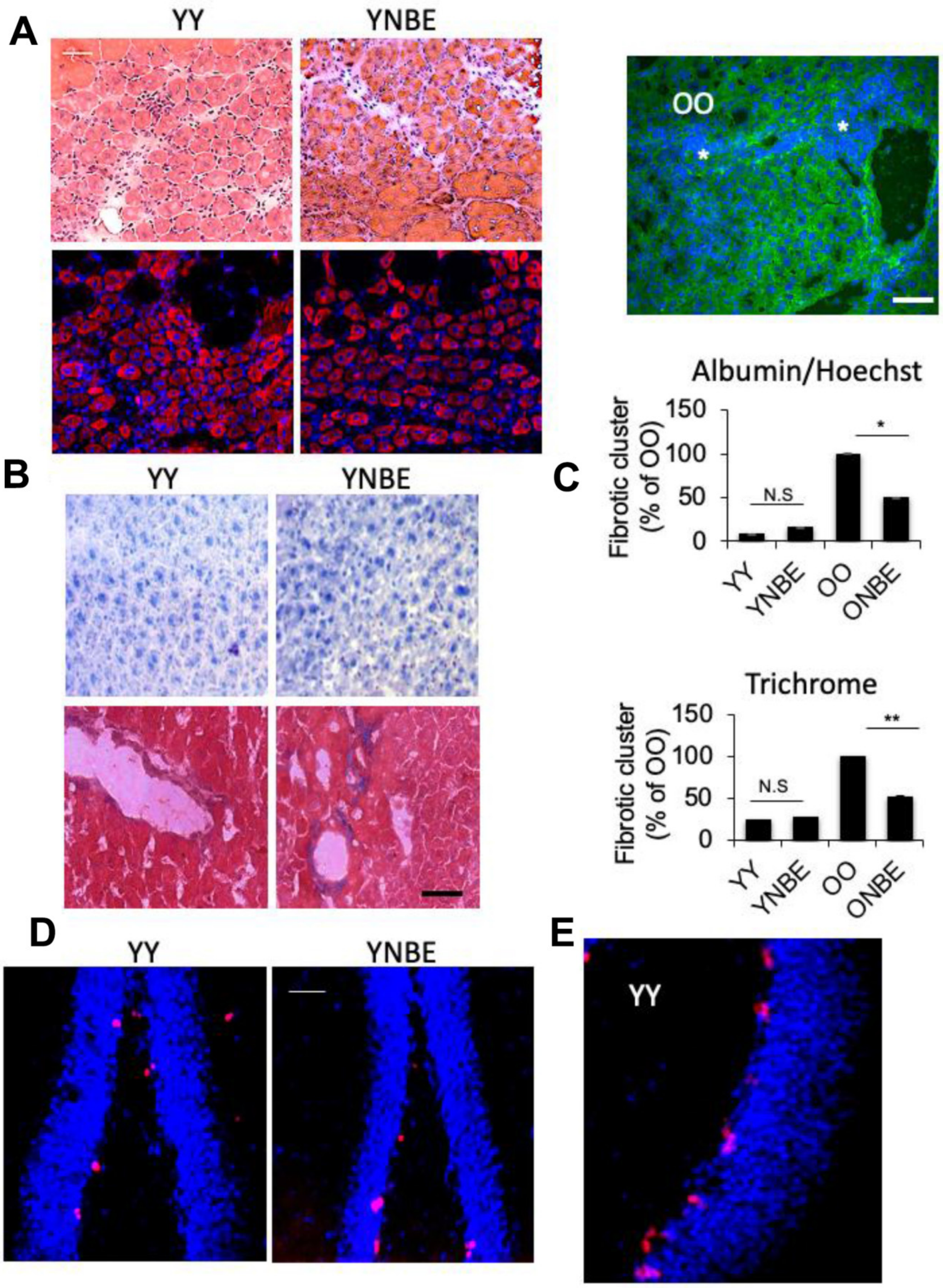

Supplementary Figure 6. Representative YY and YNBE images and comparative quantification of liver fibrosis through Trichrome versus Albumin/Hoechst immunofluorescence. (**A**) Representative H&E staining and eMyHC immunofluorescence are shown in muscle sections (10 micron). (**B**) Representative OilRed and Mason Trichrome are shown in liver sections (10 micron). (**C**) Fibrotic index of livers is quantified through Albumin/Hoechst (with representative image of that immunofluorescence, Top) and through Mason Trichrome staining. Nearly identical results are obtained with $p<0.05$. (**D**) Representative images of Dentate Gyri with Ki67+/Hoechst+ neural precursor cells visible at the SGZ. (**E**) Enlarged image that shows these cells at higher resolution. Scale bar 50μM.
