# Supplementary material for: Rejuvenation of three germ layers tissues by exchanging old blood plasma with saline-albumin
Source: Aging (Albany NY). 2020 May 30;12(10):8790–819. doi: 10.18632/aging.103418 (PMC7288913; doi:10.18632/aging.103418)
Supplement: Supplementary Table 1 [file aging-12-103418-s001..pdf]

SUPPLEMENTARY TABLE

Supplementary Table 1. Proteins that are changed by the NBE or TPE with highest statistical significance.

A. Mouse comparative proteomics: OO versus ONBE.

Category	ID	p	Significance	Change
Immune: Chemokines	Eotaxin-2	8.2E-04	***	+
	MIP-2	1.2E-03	**	+
	PF-4	1.4E-03	**	+
	CCL1 / I-309 / TCA-3	3.3E-03	**	+
	IL-16	4.3E-03	**	+
	CXCL14 / BRAK	9.1E-03	**	+
	Fractalkine	1.4E-02	*	+
	CXCR2 / IL-8 RB	2.7E-02	*	+
	CCR6	2.7E-02	*	+
	Lungkine	3.0E-02	*	+
	CCL28	3.4E-02	*	+
	TCA-3	4.0E-02	*	+
	CXCR6	4.2E-02	*	+
Immune: GF/DF	IL-3 R beta	5.2E-04	***	+
	Erythropoietin (EPO)	5.9E-03	**	+
	Lymphotoxin beta R / TNFRSF3	4.7E-02	*	+
	IL-21	5.2E-02	*	+
Tissue Homeostasis	TIMP-2	6.0E-04	***	+
	VCAM-1	8.0E-03	**	+
Growth Factors	BTC (Betacellulin)	6.3E-03	**	+
	Prolactin	9.2E-03	**	+
	Osteoactivin / GPNMB	2.0E-02	*	+
	GDF-5	2.4E-02	*	+
Pathways	IGFBP-2	3.2E-03	**	+
Other	AgRP	3.5E-03	**	+
	Urokinase	9.9E-03	**	+
Immune: Cell-fate	TWEAK R / TNFRSF12	6.7E-03	**	+
	LIGHT / TNFSF14	1.4E-02	*	+
	TNF-beta / TNFSF1B	2.6E-02	*	+
Angiogenic	TCCR / WSX-1	2.5E-02	*	+
	Angiopoietin-like 3	3.1E-02	*	+
Immune: Interleukins	IL-1 RII	2.8E-02	*	+

B. YY versus OO.

ID	p	Change
IL-20 R alpha	1.8E-02	+
Fas Ligand	1.8E-02	+
FGF-21	2.1E-02	+
IL-10 R alpha	2.1E-02	+
G-CSF	2.3E-02	+
MIP-1 gamma	2.5E-02	+

Axl	2.5E-02	+
CD27 Ligand / TNFSF7	2.6E-02	+
CD40	3.6E-02	+
EGF R	3.7E-02	+
FADD	3.8E-02	+
L-Selectin	4.2E-02	+
E-Selectin	4.5E-02	+

C. O pre TPE versus O after TPE

Category	ID	p	Significance	Change
Pathways	IFN-alpha / beta R1	6.0E-04	***	-
	Siglec-9	2.3E-03	**	+
	BMPR-IB / ALK-6	6.9E-03	**	+
	TRAIL / TNFSF10	1.4E-02	*	-
	TMEFF1 / Tomoregulin-1	1.4E-02	*	+
	Follistatin	1.5E-02	*	+
	VEGI / TNFSF15	1.9E-02	*	-
	Glut2	2.2E-02	*	+
	IL-12 R beta 1	2.3E-02	*	-
	ICAM-1	2.5E-02	*	-
	Follistatin-like 1	2.7E-02	*	+
	Glypican 5	4.0E-02	*	+
	Endothelin	4.3E-02	*	+
Tissue Homeostasis	Thrombospondin-2	5.4E-05	***	+
	uPA	5.8E-03	**	+
	Ubiquitin+1	1.1E-02	*	+
	NrCAM	1.5E-02	*	+
	Kininostatin / kininogen	2.3E-02	*	+
	Lipocalin-2	2.7E-02	*	+
	MMP-9	2.9E-02	*	+
GFs & Their Receptors	FGF R4	2.0E-03	**	+
	BDNF	2.0E-03	**	+
	IGF-I	9.6E-03	**	-
	IGF-II	1.1E-02	*	+
	CNTF	2.0E-02	*	+
Immune: Interleukins	IL-6	1.5E-03	**	-
	IL-1 ra	6.3E-03	**	+
	IL-17D	6.8E-03	**	+
	IL-31	1.3E-02	*	-
	IL-1 F10 / IL-1HY2	3.4E-02	*	+
	IL-17C	4.2E-02	*	+
Immune: Chemokines	CXCR2 / IL-8 RB	1.0E-03	**	+
	CCR9	3.4E-03	**	+
	CCR7	6.3E-03	**	+
	MIP-1a	1.6E-02	*	+
	MIP 2	1.8E-02	*	+
	PF4 / CXCL4	2.6E-02	*	+

	CXCR1 / IL-8 RA	3.5E-02	*	+
	TECK / CCL25	3.9E-02	*	-
Angiogenic	Angiopoietin-2	1.1E-02	*	+
	Angiogenin	1.3E-02	*	+
	ROBO4	1.7E-02	*	+
	Epiregulin	2.0E-02	*	-
	VEGF	2.0E-02	*	+
	Erythropoietin	2.7E-02	*	+
	VEGF-C	3.6E-02	*	-
	Angiostatin	4.4E-02	*	+
Immune: Adaptive	TRADD	1.3E-02	*	+
	SIGIRR	2.9E-02	*	+
Immune: GF/DF	M-CSF	1.2E-02	*	+

The results of comparative proteomics (described in main text) were analyzed to identify the proteins which differ the most (with highest statistical significance) between the OO vs ONBE (**A**), YY vs OO (**B**) and O pre TPE vs O after TPE (**C**). The names and categories of the proteins, as well as the p-values and direction of the change (+ means upregulated, - means down-regulated) are shown.
